# Socioeconomic differences in limited lung function: a cross-sectional study of middle-aged and older adults in Germany

**DOI:** 10.1186/s12939-024-02224-1

**Published:** 2024-07-10

**Authors:** Johannes Beller, Batoul Safieddine, Stefanie Sperlich, Juliane Tetzlaff, Siegfried Geyer

**Affiliations:** Hannover Medical School, Center for Public Health and Health Care, Medical Sociology Unit, Carl-Neuberg-Str. 1, Hannover, 30625 Germany

**Keywords:** Lung function, Functional limitations, Morbidity, Social inequality

## Abstract

**Background:**

Limited lung function represents a serious health impairment. However, studies investigating social inequalities in limited lung function are rare. Thus, the current study investigates which socioeconomic groups are the most affected by overall limited lung function and severely limited lung function.

**Methods:**

Data from the population-based German Aging Survey were used (*N* = 4472), with participants being 40 + years old. Lung function was assessed by the peak flow test. Education, income, and occupational prestige were used as socioeconomic indicators.

**Results:**

We found that overall limited lung function was highly prevalent across the whole sample, with about 33% (Women: 35%; Men: 30%) having overall limited lung function and 8% (Women: 7%; Men: 8%) having severely limited lung function. Socioeconomic differences in limited lung function emerged for all three indicators, education, income, and occupational prestige, in both men and women in single effect analyses. These differences persisted for occupational prestige and income when controlling for all indicators simultaneously.

**Conclusions:**

Thus, overall and severely limited lung function are highly prevalent health conditions. Men and women with a low occupational position and those with low income are the most affected. Socioeconomic indicators cannot be used interchangeably when studying health inequalities in lung functioning. Occupational hazards and physical working conditions are likely to constitute major risks of health inequalities in limited lung functioning and should be investigated as such by future studies.

Lung function is seen as an important indicator of physical functioning and healthy aging [1–3]. Conversely, an impaired lung function may result from cumulative exposures to toxins like tobacco smoke, fumes at work, and respiratory infections, as well as a reduced general physical functioning. Moreover, lung function is impaired in many diseases, such as COPD and Asthma and is often used as the central endpoint in clinical trials [4, 5]. Several recent studies have estimated high prevalences of limited lung function in the general population: For example, based on the US Health and Retirement Study a prevalence of 27% of limited lung function measured by reduced peak flow was found in their population-based sample of middle-aged and older adults [4]. In another study based on data from the US, 20% of participants among the general population were found to have limited lung function; among older adults this prevalence increased to 40% [6]. Prevalence of chronic lung diseases is reported to be lower but still comparatively high in the general population, with for example 5–12% of middle-aged and older German adults reporting having COPD [7].

## Socioeconomic differences

However, the relatively high prevalence of lung function impairment is not distributed equally among different socioeconomic groups [8, 9]. Socioeconomic inequalities exist in morbidity, with individuals from lower socioeconomic status groups typically being more strongly affected [10–12]. Limited lung function can be seen both from the causal and the selection theory of social inequalities. According to the causal theory, lower socioeconomic status leads to worse health outcomes such as limited lung function; according to the selection theory, worse health outcomes also lead to a lower socioeconomic status [11]. Previous studies have found supportive evidence for both perspectives, albeit in relation to other lung-related health outcomes [13, 14]. Additionally, gender may play an important role in shaping the socioeconomic inequalities in limited lung function, as it may influence the exposure and vulnerability to socioeconomic risk factors [15].

Although education, occupation and income are often used interchangeably when investigating social inequalities in health, research suggests that these variables measure different aspects of one's socioeconomic status [10, 16, 17]. Accordingly, evidence suggests that even when correlated, each of these three indicators influences health differently, following its own causal mechanisms [18]. Education enhances psychosocial resources including the knowledge to adopt healthy lifestyles, income as a material resource potentially enables a healthier lifestyle, and one’s occupation provides exposure to work resources and work hazards like fumes and dust [16, 19]. For example, Geyer and colleagues [17] investigated the individual and combined effects of education, income and occupation on all-cause mortality, myocardial infarction and type 2-diabetes. The authors found differences in the strength of associations between socioeconomic indicators, which also differed according to the examined outcome. Thus, considering all three indicators simultaneously is essential for obtaining a comprehensive picture of socioeconomic inequalities in limited lung function. Doing so will help to identify important target groups and starting points for planning preventive interventions.

### Aim of the study

Population-based studies examining socioeconomic differences in limited lung function are lacking. Previous studies did not consider the individual and simultaneous effects of the three socioeconomic indicators (e.g., [8, 9]). Additionally, most previous studies were conducted in an US-American context and cross-national generalizability of findings remains unclear. Thus, information is missing on which socioeconomic indicators might be most strongly and consistently associated with limited lung function. The current study aims to help fill this gap in the literature. It contributes to the literature by examining socioeconomic differences in limited lung function in a population-based German sample. Thereby, vulnerable groups for limited lung function are also identified, which might constitute the basis for additional prevention and intervention efforts. We ask: “How are education, income and occupational prestige related to limited lung function?”.

## Methods

### Sample

Data were drawn from the public release of the German Aging Survey [20]. The German Aging Survey (Deutscher Alterssurvey; DEAS) is a cohort-sequential population-based study on Germans aged 40 years and older that is provided by the Research Data Center of the German Center of Gerontology [21]. For the German Aging Survey participants are drawn randomly by probability sampling. Additionally, participants from previous waves are re-contacted. The DEAS baseline samples are based on a two-stage sampling methodology, that involves a random sample of municipalities in Germany and a random sample of individuals within those municipalities. In each baseline year, the local population registries of the previously randomly selected 290 municipalities provide the basis used to sample the population of people living in the community in private households, aged between 40 and 85 years. The oldest age group, along with the group of men and of East Germans, are oversampled to ensure that there is a reasonable number of participants in these demographic subgroups, such as men in old age living in East Germany. All interviews are conducted face-to-face in the participant’s residence. All procedures are in accordance with German law and the ethical standards of the 1964 Helsinki declaration and its later amendments. The 2014 wave was the most recent wave, as of writing the manuscript, in which a population-based sample was drawn and lung function was measured. Thus, we used data from all baseline participants in 2014 (*N* = 6001). After excluding participants with missing values listwise (about 25% of the sample), a final sample with *N* = 4472 participants resulted.

### Measures

#### Limited lung function

Lung function was measured via the peak flow method, as the maximum expiratory flow [22]. The peak flow has been shown to be a useful approximation of the general lung function of individuals when full spirometry is unavailable, as is typically the case in large population-based studies [3, 23, 24]. Lung function measurements were performed during the face-to-face interviews using a Mini-Wright Peak Flow Meter. For each participant, two measurements of lung function were performed at intervals of at least 30 s. The maximum test results were recorded and used as measures of lung function in the current study. In line with the literature, equations from European spirometry guidelines were used to calculate percentage of predicted lung function to identify cases with overall and severely limited lung function [25]. As in most previous studies, observed peak expiratory flow rates of less than 80% of that predicted based on reference values were classified as having overall limited lung function (e.g., [26–28]). Additionally, to examine the robustness of the findings according to limitation severity, participants with peak expiratory flow rates of less than 50% of predicted were classified as having severely limited lung function [29]. Thus, limited lung function is operationalized via two indicators: Overall limited lung function (1 = “peak flow < 80%” vs. 0 = “peak flow >  = 80%”), and severely limited lung function (1 = “peak flow < 50%” vs. 0 = “peak flow >  = 50%”). Therefore, the indicators of overall limited and severely limited lung function are not mutually exclusive as all those with severely limited lung function also have overall limited lung function. Severely limited lung function rather represents a more severe condition.

#### Socioeconomic status

Socioeconomic status was operationalized by means of three socioeconomic indicators [17]: education, income and occupational prestige. Education was measured by the highest obtained school leaving qualification, with qualifications up to lower secondary level coded as “Low”, up to higher secondary as “Intermediate” and beginning from a general qualification for university entrance up to completed tertiary education as “High”. Income was based on the participants’ self-report of their total monthly net household income in euros. This included all income types like wages, salaries, self-employment income, and any form of retirement benefits. To improve comparability between studies and countries, the percentage of the equivalized income was calculated by adjusting the net income according to household size and then calculating the income relative to the mean equivalent income in the general German population [21]. Our income variable thus shows the individual income position in percentage points of the mean equivalent income of the German population. It was classified in three groups: “Low” (income < 80% of the mean equivalent income in the population), “Intermediate” (income >  = 80% and <  = 120% of the mean equivalent income in the population), and “High” (income > 120% of the mean equivalent income in the population). Finally, occupational prestige was assigned based on the actual or last occupation of the respondent and the current partner or former spouse in accordance with the procedures of the SIOPS Standard International Occupation Prestige-Scale [30, 31] considering only the higher value of both persons. Prestige values were categorized following a 5-point scale ranging from “Very Low” (prestige scores from 6 to 32 mainly covering unskilled, semi-skilled, manual work), “Low” (prestige scores from 33 to 41 mainly covering undemanding, routine jobs), “Intermediate” (prestige scores from 42 to 50 mainly covering work involving demanding tasks following general instructions), and “High” (prestige scores from 51 to 63 mainly covering work involving independent tasks in responsible job and with limited responsibility for personnel) to “Very High” (prestige scores from 64 to 78 mainly covering work involving far-reaching leadership tasks and decision-making powers) as proposed by Hoffmeyer-Zlotnik and Warner [32].

Additionally, as a robustness analysis occupational group instead of occupational prestige will be used as the occupation-related socioeconomic indicator. In this case, occupations were classified according to the International Standard Classification of Occupations (ISCO) as being high skilled white collar (WC-HS; ISCO major groups 1, 2 and 3), low skilled white collar (WC-LS; ISCO major groups 4 and 5), high skilled blue collar (BC-HS; ISCO major groups 6 and 7), or low skilled blue collar (BC-LS; ISCO major groups 8 and 9). In another robustness analysis wealth was used as an economic indicator instead of income. Wealth was based on the participants’ self-report of their total net household wealth in Euros. This included all assets, only excluding real estate. The wealth variable was classified into three groups: “Low” (total wealth <  = 5.000€), “Intermediate” (total wealth > 5.000€ & < 100.000€), and “High” (total wealth >  = 100.000€). In a further robustness analysis three additional covariates were considered: Network size was operationalized based on the self-reported count of important persons with regular contact from 0 (no one reported) up to 9 (9 or more persons reported). Migration status was operationalized based on self-reported migration background with or without personal migration experience (coded as 1) or no self-reported migration background (coded as 0). Finally, the number of chronic diseases was operationalized as the number of self-reported chronic diseases based on a list of 18 distinct diseases, including for example diabetes, myocardial infarction, chronic lung disease and cancer.

### Data analysis

First, we employed descriptive statistical methods to summarize the characteristics of our study sample. This included calculating means and standard deviations for continuous variables and percentages for categorical variables. Then, correlation (Spearman) and logistic regression analyses were conducted to investigate the associations of socioeconomic indicators with the prevalence of limited lung function. Two types of logistic regression models with varying model complexity were calculated. In the first model (single effect model), only the respective sociodemographic indicator and age were included, thus conducting separate analyses for all social inequality indicators. In this case, “gross” effects of single socio-economic indicators are calculated. Furthermore, logistic regression analyses were calculated which included education, income, occupational prestige, and age simultaneously, thus estimating the unique contribution of social inequality when controlling for other socioeconomic indicators (simultaneous effect model). In this case, “net” effects of socio-economic indicators are calculated. By calculating both models, one can estimate the degree to which the observed socioeconomic differences for individual indicators (single effect model) can be explained by their interrelationships with the other socioeconomic indicators (simultaneous effect model). We stratified all analyses by gender, as one might expect gender differences in associations between the socioeconomic status and health outcomes.

Additionally, several robustness analyses were conducted. In one robustness analysis occupational group instead of occupational prestige is used as the occupation-related socioeconomic indicator. In another robustness analysis, we also included chronic diseases, network size and migration status as additional covariates in the logistic regression models. In another robustness analysis wealth was used as an economic indicator instead of income. As another robustness analysis weighting was used. The DEAS data provide sampling weights that adjust for the multistage sampling design and nonresponse. We applied these weights in a robustness analysis to account for the potential effects of the sampling scheme. As a further robustness analysis, we also calculated the prevalence ratio (PR) for each socioeconomic indicator using a log-binomial regression model, which has been recommended in the literature for highly prevalent outcomes [33]. As the final robustness analysis, we also performed a *p*-value based recursive partitioning analysis to exploratorily identify possible interactions between the predictors in determining vulnerable groups in limited lung function [34]. This method uses a significance test approach to split the data into homogeneous subgroups based on the predictors, without the need to specify interaction terms a priori.

## Results

As depicted in Table 1, participants were on average 62.08 (*SD* = 11.66) years old, with 47% being female. On average, 33% of participants (women: 36%; men: 30%) were classified as having an overall limited lung function (peak flow < 80%), and 8% (women: 7%; men: 8%) of all participants were classified as having a severely limited lung function (peak flow < 50%). As depicted in Table 2, participants with an overall limited lung function were older, more likely to be female, had a lower educational level, lower income level and a lower occupational prestige than participants without limited lung function in univariate analyses. Similarly, as depicted in Table 3, participants with a severely limited lung function were also older, had a lower educational level, lower income level and a lower occupational prestige than participants without limited lung function in univariate analyses. Education, Income and Occupation correlated moderately with each other (0.41 ≤ *r* ≤ 0.49; Table 4 in Appendix).

**Table 1:** Limited lung function, socioeconomic status, and demographic variables in the sample (*N* = 4472)

Variable	Stratified by Gender		
	Overall	Male	Female
n	4472	2342	2130
Overall Limited Lung Function (Peak Flow < 80% Predicted) (%)	32.5	29.8	35.4
Severely Limited Lung Function (Peak Flow < 50% Predicted) (%)	7.5	7.6	7.4
Age (mean (SD))	62.08 (11.66)	62.76 (11.57)	61.33 (11.71)
Female (%)	47.6	0.0	100.0
Education (%)			
High	29.6	33.3	25.5
Intermediate	32.8	28.8	37.2
Low	37.7	37.9	37.4
Income (%)			
> 120%	30.5	32.4	28.4
80%-120%	31.7	32.1	31.3
< 80%	37.8	35.5	40.4
Occupational Prestige (%)			
Very High	17.5	18.1	16.9
High	25.5	25.8	25.1
Medium	36.8	36.0	37.7
Low	11.9	10.9	12.9
Very Low	8.4	9.3	7.4

**Table 2:** Socioeconomic status and demographics according to overall limited lung function in the sample (*N* = 4472)

	Stratified by Overall Limited Lung Function		
	Not Limited (PF > = 80%)	Limited (PF < 80%)	*p*
N	3019	1453	
Age (mean (SD))	60.08 (11.18)	66.23 (11.52)	< .001
Female (%)	45.5	52.0	< .001
Education (%)			< .001
High	33.4	21.6	
Intermediate	35.2	27.7	
Low	31.4	50.6	
Income (%)			< .001
> 120%	35.3	20.5	
80%-120%	32.3	30.5	
< 80%	32.4	49.0	
Occupational Prestige (%)			< .001
Very High	19.3	13.6	
High	27.4	21.5	
Medium	35.7	39.0	
Low	10.6	14.5	
Very Low	7.0	11.4	

*p*-values were calculated based on t and χ2 tests

**Table 3:** Socioeconomic status and demographics according to severely limited lung function in the sample (*N* = 4472)

	Stratified by Overall Limited Lung Function		
	Not Limited (PF > = 50%)	Limited (PF < 50%)	*p*
N	4137	335	
Age (mean (SD))	61.48 (11.50)	69.51 (10.94)	< .001
Female (%)	47.7	46.9	.815
Education (%)			< .001
High	30.4	18.9	
Intermediate	33.9	18.9	
Low	35.7	62.2	
Income (%)			< .001
> 120%	31.7	15.8	
80%-120%	32.0	28.1	
< 80%	36.3	56.1	
Occupational Prestige (%)			< .001
Very High	18.1	10.4	
High	26.0	19.1	
Medium	36.5	41.2	
Low	11.5	15.8	
Very Low	8.0	13.4	

*p*-values were calculated based on t and χ2 tests

### Overall limited lung function

**Fig. 1:**
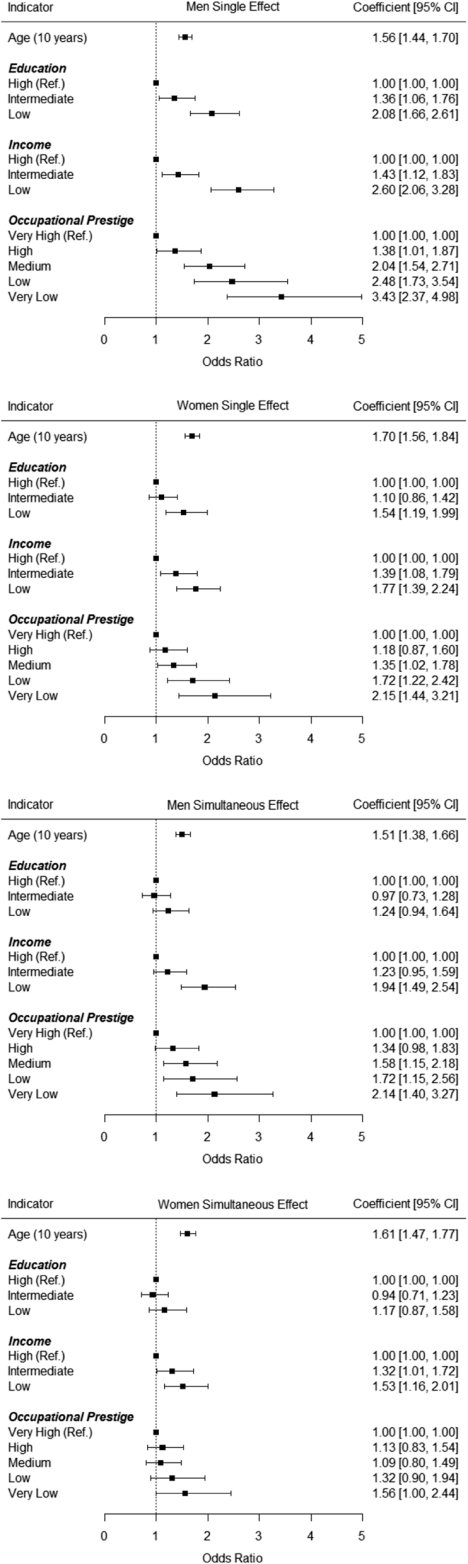
Overall Limited Lung Function: Socioeconomic Differences as Predicted by Logistic Regression. Notes. Estimates represent results from logistic regression analyses and were controlled for age (single effect models) or age and all three socioeconomic indicators (simultaneous effect models)

Next, logistic regression analyses were used to study socioeconomic differences in limited lung function, controlled for age with the socioeconomic indicators estimated separately (single effect model) and limited lung function controlled for age with all socioeconomic indicators estimated simultaneously (simultaneous effect model). As depicted in Fig. 1, being 10 years older corresponded to an increased risk of having limited lung function of *OR* = 1.56 in men and *OR* = 1.70 in women. Furthermore, in the single effect models social gradients emerged regarding each socioeconomic indicator (education, income and occupational prestige) with lower socioeconomic status levels being associated with a higher likelihood of having an overall limited lung function in both men and women. Largest effects in the single effect models emerged for occupational prestige (e.g., *OR*_*Very Low*_ = 3.43 in men and *OR*_*Very Low*_ = 2.15 in women). In the simultaneous effect models, being 10 years older corresponded to an increased risk of having limited lung function of *OR* = 1.51 in men and *OR* = 1.61 in women. Significant socioeconomic differences emerged only for income and occupational prestige but not for education, with lower socioeconomic status levels also being associated with a higher likelihood of having an overall limited lung function in both men and women. Largest effects in the simultaneous effect models also emerged for occupational prestige (e.g., *OR*_*Very Low*_ = 2.14 in men and *OR*_*Very Low*_ = 1.56 in women). Thus, socioeconomic differences in overall limited lung function corresponded to about as large an increase in risk as ageing 10 to 20 years.

The finding that mostly occupation and income, but not education, were associated with differential rates of limited lung function was also replicated in robustness analyses using occupational group instead of occupational prestige as an indicator (please see the Table 5 and Figure 3 in Appendix). As a further robustness analysis, we investigated whether the inclusion of wealth instead of income would alter the results of the main analysis. The results of this analysis are shown in Table 6 and Figure 4 in Appendix. The patterns of associations between socioeconomic indicators and limited lung function were also similar to those of the main analysis, with occupational prestige and especially wealth showing the strongest and most consistent effects. Similar results were also obtained when using weights in the regression analysis (Figure 5 in Appendix). Regarding the use of the log-binomial model, the PRs for overall limited lung function were similar to the ORs, albeit smaller in size, with lower socioeconomic status levels being associated with a higher prevalence of overall limited lung function in both men and women (Figure 6 in Appendix). For example, men with low income were 1.54 times as likely to have overall limited lung function as compared to men with high income (*PR*_*Low*_ = 1.54), and men with very low occupational prestige were 1.56 times as likely to have overall limited lung function as compared to men with very high occupational prestige (*PR*_*Very Low*_ = 1.56). Additionally, we conducted a robustness analysis to examine whether the inclusion of chronic diseases, network size and migration status as additional covariates would alter the results of the main analysis. Similar results as in the main analysis emerged but were not as statistically significant. The results of this analysis are shown in Figure 7 in Appendix. As a last robustness analysis, the results of the *p*-value based recursive partitioning analysis are shown in Figure 8 in Appendix. The analysis identified 9 subgroups of participants with different levels of overall limited lung function according to sociodemographic predictors. The most vulnerable subgroup with the highest prevalence of overall limited lung function consisted of participants aged 77 years and older with a low educational level; among comparatively younger participants, those with a lower income level of < 80% and a low education were the most affected by overall limited lung function. The least vulnerable subgroup, on the other hand, with the lowest prevalence of overall limited lung function consisted of participants aged 40–52 years who also had a high income of > 120%. Overall, the recursive partitioning results suggest that age, income and education are the most important predictors of limited lung function, and that there are significant interactions among these variables. Thus, the patterns of associations between socioeconomic indicators and limited lung function were largely similar to those of the main analysis, with substantial social inequalities being observed in limited lung function.

### Severely limited lung function

**Fig. 2:**
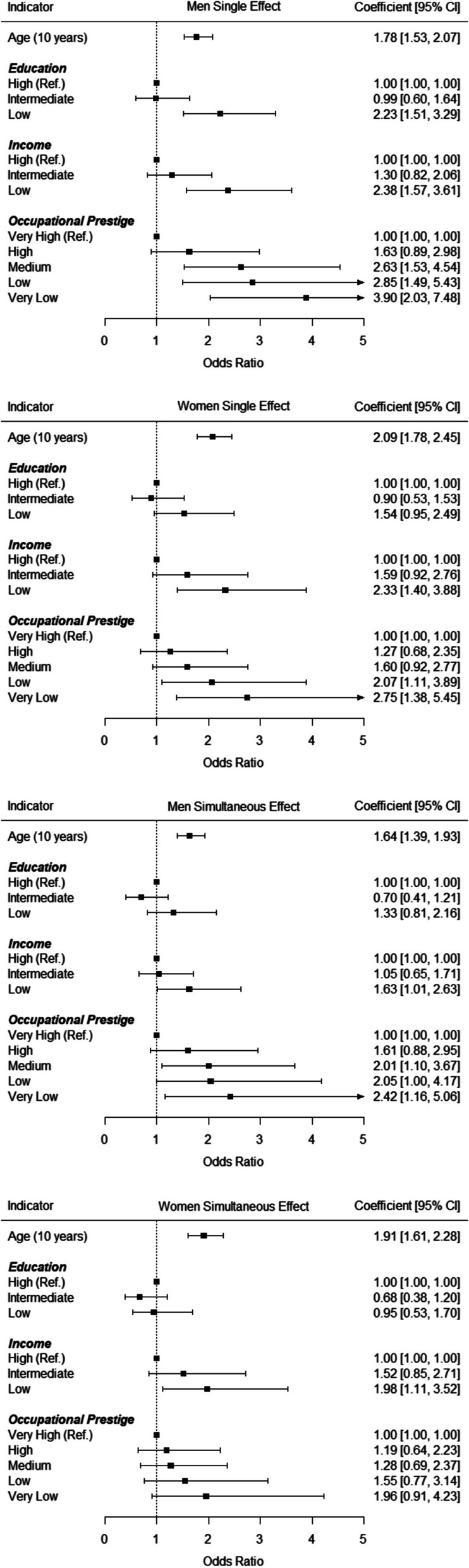
Severely Limited Lung Function: Socioeconomic Differences as Predicted by Logistic Regression. Notes. Estimates represent results from logistic regression analyses and were controlled for age (single effect models) or age and all three socioeconomic indicators (simultaneous effect models)

**Fig. 3:**
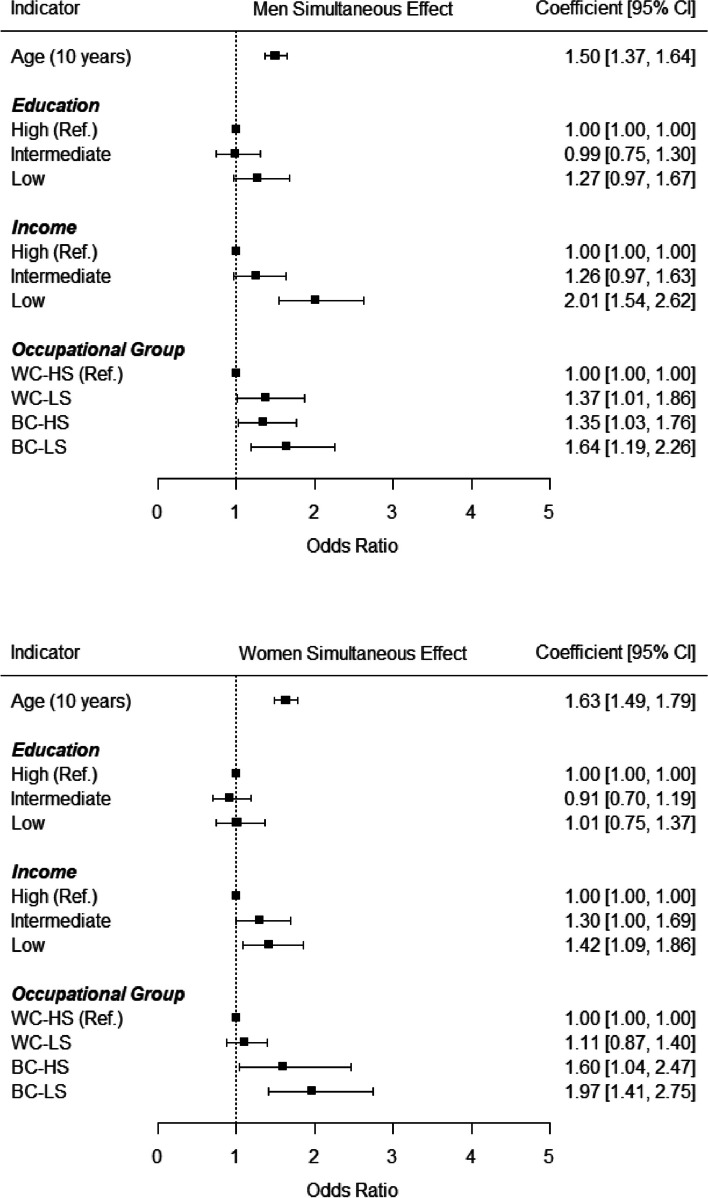
Overall Limited Lung Function: Socioeconomic Differences as Predicted by Logistic Regression using Occupational Group instead of Occupational Prestige. Notes. Estimates represent results from logistic regression analyses and were controlled for age and all three socioeconomic indicators (simultaneous effect models)

**Fig. 4:**
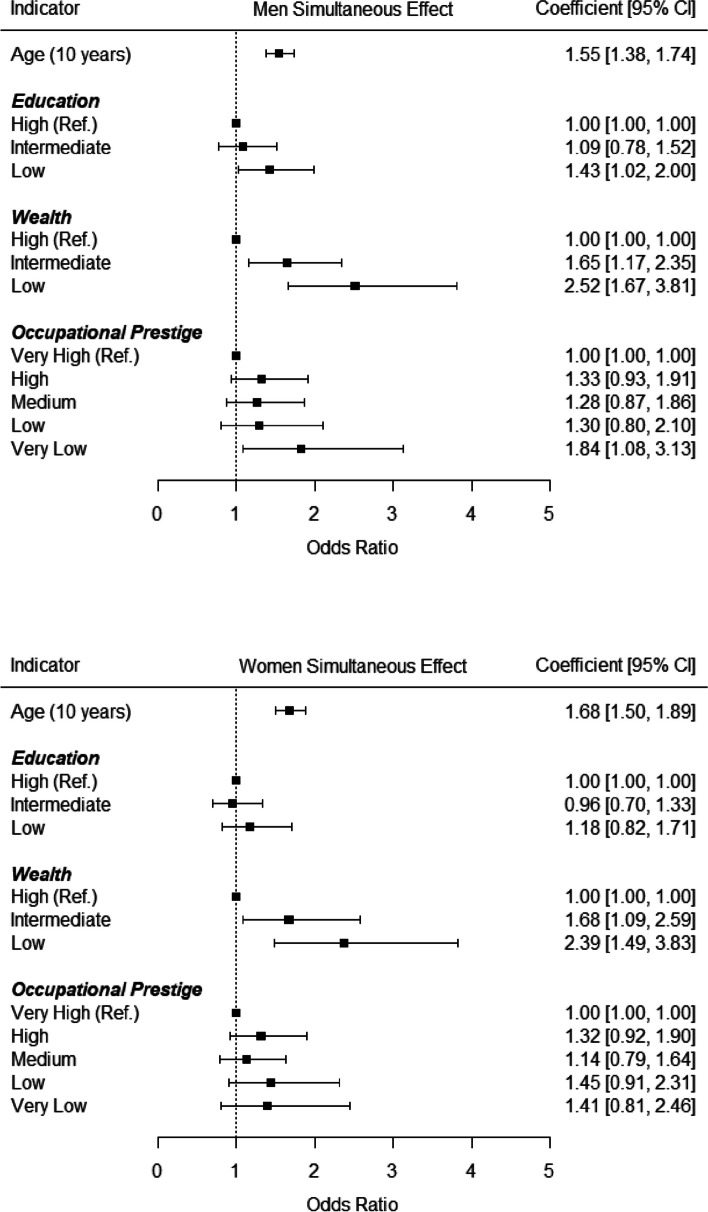
Overall Limited Lung Function: Socioeconomic Differences as Predicted by Logistic Regression using Wealth instead of Income. Notes. Estimates represent results from logistic regression analyses and were controlled for age and all three socioeconomic indicators (simultaneous effect models)

**Fig. 5:**
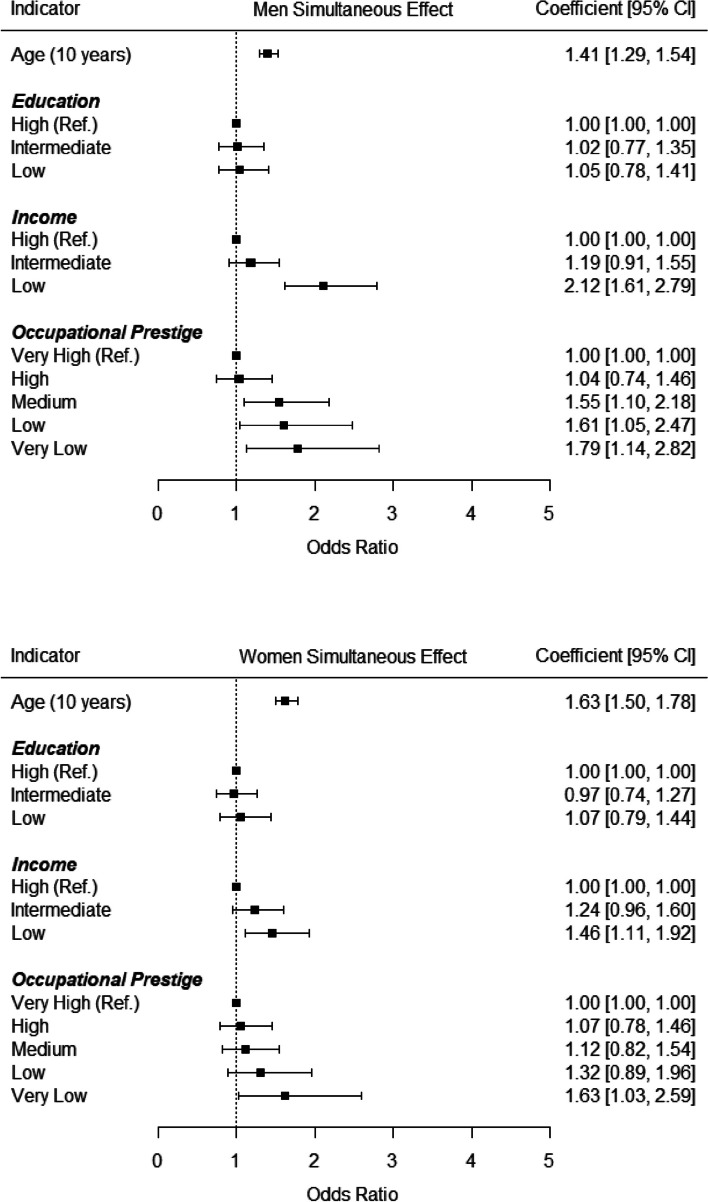
Overall Limited Lung Function: Socioeconomic Differences as Predicted by Weighted Logistic Regression. Notes. Estimates represent results from logistic regression analyses and were controlled for age and all three socioeconomic indicators (simultaneous effect models)

**Fig. 6:**
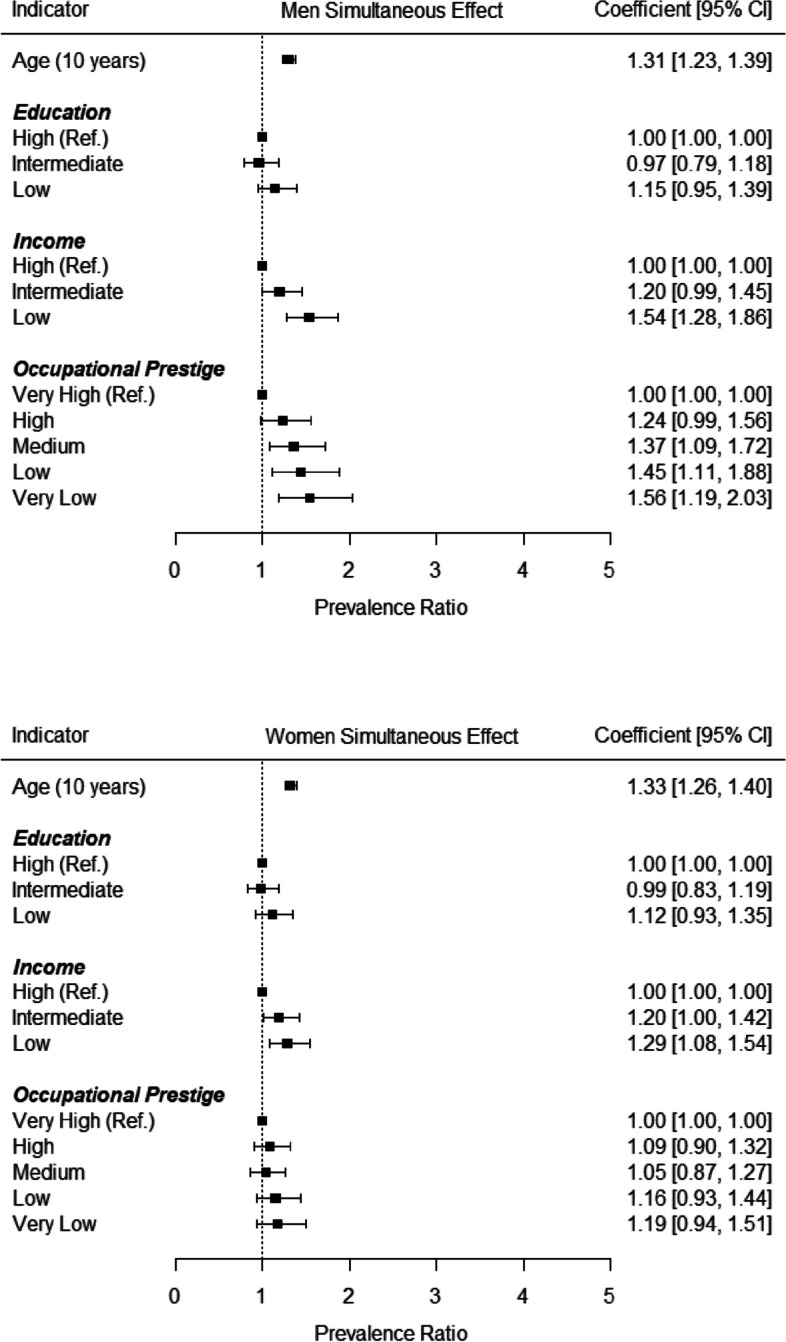
Overall Limited Lung Function: Socioeconomic Differences as Predicted by Log-Binomial Regression. Notes. Estimates represent results from log-binomial regression analyses and were controlled for age and all three socioeconomic indicators (simultaneous effect models)

**Fig. 7:**
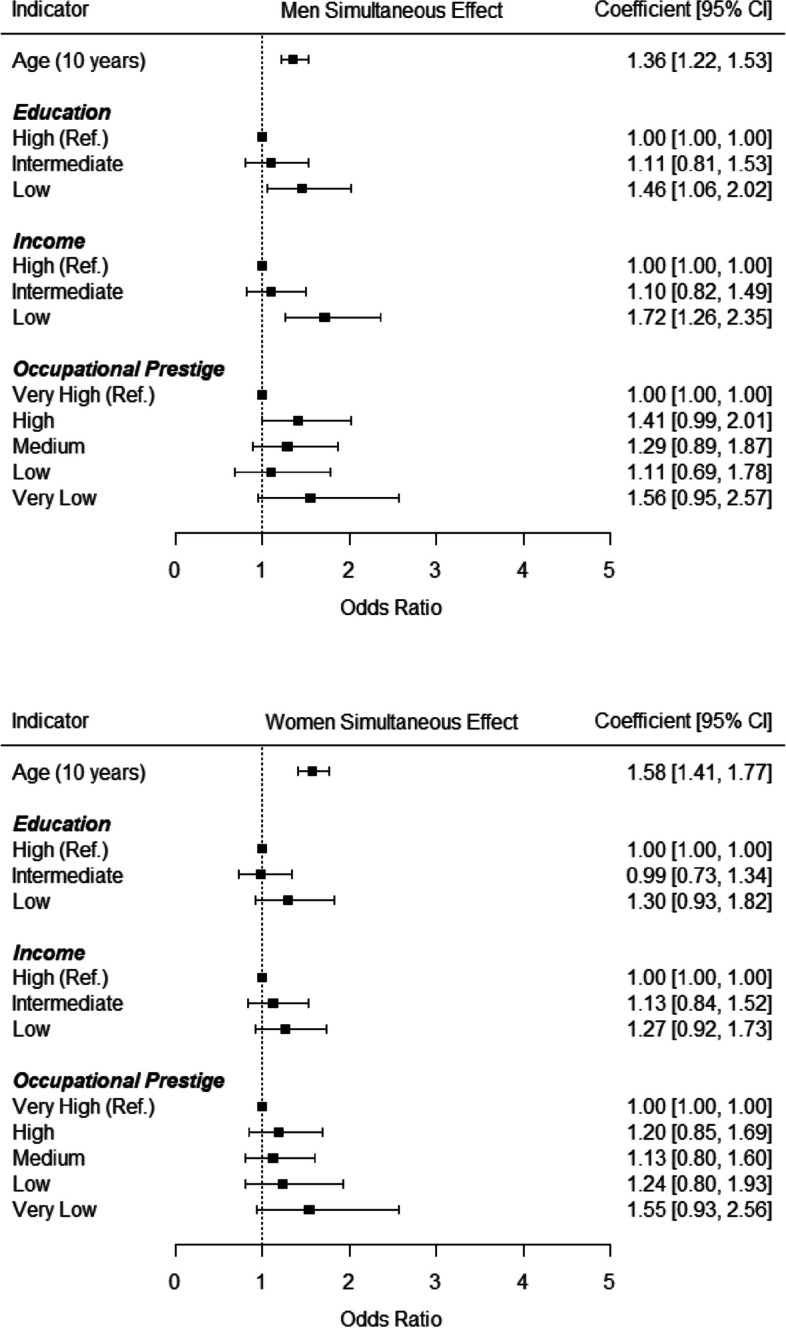
Overall Limited Lung Function: Socioeconomic Differences as Predicted by Logistic Regression Controlled for Chronic Diseases, Migration Status and Network Size. Notes. Estimates represent results from logistic regression analyses and were controlled for age and all three socioeconomic indicators (simultaneous effect models) as well as chronic diseases, migration status and network size

**Fig. 8:**
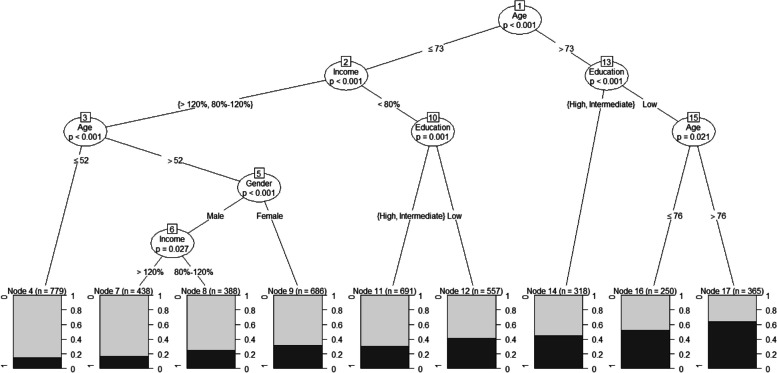
Overall Limited Lung Function: Vulnerable Groups via *P*-Value based Recursive Partitioning. Notes. Bins at the Bottom Represent Prevalence of Overall Limited Lung Function in the Respective Subgroup (dark shaded area)

Similar socioeconomic differences were found regarding severely limited lung function, as depicted in Fig. 2. In the single effect models, being 10 years older corresponded to an increased risk of having limited lung function of *OR* = 1.78 in men and *OR* = 2.09 in women. Furthermore, social gradients emerged for both men and women only for income and occupational prestige, with lower socioeconomic status levels being associated with a higher likelihood of having a severely limited lung function. Largest effects in the single effect models emerged again for occupational prestige (e.g., *OR*_*Very Low*_ = 3.90 in men and *OR*_*Very Low*_ = 2.75 in women). In the simultaneous effect models, being 10 years older corresponded to an increased risk of having limited lung function of *OR* = 1.64 in men and *OR* = 1.91 in women. Significant socioeconomic differences emerged only for income in both genders, with lower socioeconomic status levels also being associated with a higher likelihood of having a severely limited lung function. Largest effects in the simultaneous effect models emerged for occupational prestige in men (e.g., *OR*_*Very Low*_ = 2.42) and for income in women (e.g., *OR*_*Low*_ = 1.98). Thus, socioeconomic differences in severely limited lung function corresponded to about as large an increase in risk as ageing 10 to 20 years.

## Discussion

Given the importance of lung function to healthy aging and morbidity development, we examined socioeconomic differences in limited lung function according to education, income and occupation among a population-based sample of middle-aged and older adults in Germany. We found a high prevalence of overall and severely limited lung function in our study, with about a third of participants being at least moderately impaired in their lung function. Additionally, social inequalities emerged for every indicator. However, the extent to which socioeconomic differences emerged differed: Education had the smallest effect size regarding overall and severely limited lung function, in both men and women. Conversely, occupational prestige and income emerged as the indicators with larger effect sizes. These effect sizes were substantial: In the gross effect model differences up to *OR* = 3.90 emerged in men, implying that compared to participants with a very high occupational prestige participants with a low occupational prestige had up to 390% as high odds of experiencing a severely limited lung function. Even when estimating all social inequality indicators simultaneously these risks were predicted to be about as large as ageing 10–20 years. These results were consistent across various analytical approaches reported in the Appendix, indicating that socioeconomic disparities in limited lung function are robust and substantial.

These findings are likely to extend beyond Germany. Germany, like many high-income countries, experiences a well-documented demographic shift towards an aging population [35]. This shift is characterized by a higher life expectancy and a declining birth rate, resulting in a growing proportion of older individuals within the society, making studying health inequalities among the middle-aged and older population highly relevant. The morbidity patterns observed in the German population are comparable to those in other European industrialized countries, making the findings from the study also relevant for other high-income countries [36].

### Comparison with previous studies

The finding that large socioeconomic differences exist for limited lung functioning and health in general are in line with the literature [8, 9, 11, 17] and replicates previous research on lung-related diseases (e.g., [37, 38]). Going beyond previous studies, we investigated the individual and combined effects of education, income and occupational prestige, which had not been examined so far. It was found that all indicators exhibited effects when analysed separately. However, when analysed together, the effect of education became non-significant in most cases. Additionally, in both the single and simultaneous effect models socioeconomic differences appeared generally largest and most significantly for occupational prestige. This suggests that all three indicators exhibit common as well as individual effects on the risk of limited lung function. Likely, as shown in the literature, the three indicators exhibit partly different causal or influential pathways in determining health outcomes, including health competencies, material resources and environmental hazards [16, 17]. The fact that the largest effects emerged for occupational prestige suggests that risk and protective factors immanent to one’s occupation are most strongly associated with limited lung function on the population level, including occupational risks like exposures to fumes and dust and occupational rewards like social prestige and control [39–41]. We have included some variables—chronic diseases, network size and migration status—as covariates in a robustness analysis, which may function as a first step to partially explain the observed social inequalities, and which should be further investigated by future studies [42].

Another aspect that deserves attention is the role of wealth as an indicator of socioeconomic status. In a robustness analysis, we found that wealth seemed to have particularly strong associations with limited lung function. This might suggest that wealth better captures some aspects of socioeconomic status important to lung function, such as the cumulative effects of socioeconomic status over the life course and the effects of intergenerational transmission of wealth. Wealth might also reflect the long-term consequences of limited lung function on economic opportunities and social mobility. In line with this hypothesis, previous studies have shown that wealth constitutes an important determinant of health and health inequalities in middle aged and older adults [43, 44]. Future studies should consider further investigating wealth as an indicator of socioeconomic status and investigate its potential mechanisms in relation to lung function and other health outcomes.

### Public health implications

From an applied perspective, the high observed prevalence of limited lung function seems concerning. Assuming a population of 48 million adults over the age of 40 in Germany, our results suggest that about 15 million German adults above 40 would suffer from a overall limited lung function of which about 4 million are severely limited. However, this seemingly high prevalence of limited lung function is also in line with previous studies were prevalences up to 40% among older adults were reported in the US [6]. Thus, pulmonary diseases and limited functioning constitute one of the leading causes of morbidity and mortality in Germany [45]. From a public health perspective, the high level of health inequalities represents a further issue that should become a stronger focus in the future. The strength and consistency of the inequalities found with regard to occupation raises the question of whether people in low-prestige, high-strain occupations are ageing healthily enough to be able to meet the growing demands in the course of extending working lives. This emphasises the importance of measures aimed at improving lung health. Our study suggests that there is great potential in the area of occupational health and workplace prevention and that people in high-strain and low-prestige occupations could benefit most. Special prevention efforts should thus be applied to groups vulnerable to limited lung function, especially those having a low occupational prestige and a low income.

### Limitations

The results of the current study are subject to limitations. Most importantly, peak flow represents only one specific indicator of lung functioning that is favourably used in large epidemiological studies due its ease of measurement [1, 2]. While peak flow strongly correlates with other indicators of lung functioning and pulmonary disease severity, it cannot be used interchangeably with other indicators like the forced expiratory volume and forced vital capacity, especially in clinical diagnosis [23, 24, 46]. Furthermore, the sample did not include institutionalized older adults and thus likely underestimates the true level of limited lung function in the population, especially because previous studies have shown health biases in survey samples [47]. Furthermore, although our study controlled for some covariates in a robustness analysis, it did not consider other factors that may also contribute to socioeconomic inequalities in limited lung function, such as health behaviors [48], occupational hazards [49] and socio-political factors [50]. Future research should explore the role of these aspects in shaping lung health across different socioeconomic groups. Additionally, the study used cross-sectional data and thus cannot determine the causal direction of the association between lung function and socioeconomic status. Longitudinal studies are needed to examine whether limited lung function precedes or follows a low socioeconomic status, or whether there are bidirectional influences over the life course. Another limitation of our study might be that we used logistic regression and the OR as the measure of association, which may inaccurately estimate the strength of the association when the outcome is common in the population [51]. However, the OR is a widely used and accepted measure of association in epidemiological studies, and similar results to the OR were found in a robustness analysis using prevalence ratios via log-binomial regression, indicating that our findings are robust and consistent to data analytical choice. Additionally, similar results were found for overall limited lung function and severely limited lung function, emphasizing the robustness of the results. Nonetheless future studies might use additional methods to study social inequalities in limited lung function in greater depth. Finally, comparing the contribution of education, income and occupation might be complicated due to their operationalization: While the first two indicators are mostly operationalized as comprising three categories, occupational prestige is typically operationalized on the basis of five categories [32]. However, it must also be noted that the importance of occupation in inequalities in limited lung function were replicated in a robustness analysis. Nonetheless, clearly more studies are needed to investigate social inequality in lung functioning using population-based data. Future studies could also perform formal statistical tests to compare the pattern of associations of the socioeconomic indicators across gender and lung function categories, as this might reveal important differences in the mechanisms underlying the social gradients between genders.

Another potential limitation of our study is the validity of the income information of the participants. We used self-reported household income as a measure of income, which may be subject to recall bias or social desirability bias. However, previous studies have shown that self-reported income seems generally reliable and valid in population-based surveys [52]. Moreover, we adjusted the income according to household size and calculated the percentage of the equivalized income relative to the equivalent income in the general German population, which improves the comparability and representativeness of our income measure. Therefore, although future studies might use other income operationalizations, we believe that our income measure is sufficiently valid and robust for the purpose of our study.

## Conclusions

In conclusion, large socioeconomic differences exist in overall and severely limited lung function in Germany, with groups with a lower socioeconomic status being more strongly affected. However, education, income and occupational prestige were differentially associated with limited lung function, with the largest differences being observed regarding occupational prestige and income. Thus, socioeconomic indicators cannot be used interchangeably when studying health inequalities in lung functioning. Occupational hazards and rewards are likely to constitute major causes of health inequalities in limited lung functioning and should thus be further investigated by future studies.

## Data Availability

The data are available from the German Centre of Gerontology (https://www.dza.de/en/research/fdz/german-ageing-survey/deas-documentation/doi-deas).

## Appendix

**Table 4:** Correlations (Spearman) of education, income and occupational prestige

	(1)	(2)	(3)
1. Education	-		
2. Income	0.41***	-	
3. Occupational Prestige	0.49***	0.45***	-

^***^ = *p* < .001

**Table 5:** Limited lung function, ISCO occupational group, and demographic variables in the sample (*N* = 4403)

Variable	Stratified by Gender		
	Overall	Male	Female
n	4403	2318	2085
Overall Limited Lung Function (Peak Flow < 80% Predicted) (%)	32.4	29.6	35.5
Severely Limited Lung Function (Peak Flow < 50% Predicted) (%)	7.5	7.6	7.3
Age (mean (SD))	62.03 (11.63)	62.75 (11.58)	61.23 (11.64)
Female (%)	47.4	0.0	100.0
Education (%)			
High	29.5	33.3	25.4
Intermediate	32.9	28.8	37.5
Low	37.6	38.0	37.2
Income (%)			
> 120%	30.5	32.5	28.3
80%-120%	31.7	32.0	31.4
< 80%	37.7	35.5	40.2
Occupational Group (%)			
WC-HS	52.9	54.4	51.2
WC-LS	21.3	12.3	31.2
BC-HS	14.1	21.7	5.6
BC-LS	11.8	11.5	12.1

**Table 6:** Limited lung function, wealth, and demographic variables in the sample (*N* = 2964)

Variable	Stratified by Gender		
	Overall	Male	Female
N	2964	1543	1421
Overall Limited Lung Function (Peak Flow < 80% Predicted) (%)	29.8	27.5	32.2
Severely Limited Lung Function (Peak Flow < 50% Predicted) (%)	6.3	6.3	6.3
Age (mean (SD))	62.11 (11.44)	63.12 (11.43)	61.01 (11.35)
Female (%)	47.9	0.0	100.0
Education (%)			
High	33.5	34.3	32.5
Intermediate	33.7	29.0	38.8
Low	32.8	36.6	28.6
Wealth (%)			
High	15.2	18.7	11.5
Intermediate	60.7	61.0	60.4
Low	24.1	20.3	28.1
Occupational Prestige (%)			
Very High	19.8	20.5	18.9
High	27.4	28.5	26.2
Medium	35.3	33.6	37.1
Low	10.8	10.3	11.3
Very Low	6.8	7.1	6.5
